# Emphysematous Prostatic Abscess: A Case Report and Literature Review

**DOI:** 10.1002/ccr3.70544

**Published:** 2025-06-04

**Authors:** Xiaomin Yi, Yunqiang Shao, Jiexiu Zhang, Ruoyun Tan

**Affiliations:** ^1^ Department of Urology First Affiliated Hospital of Nanjing Medical University Nanjing Jiangsu China; ^2^ Department of Urology Nanjing Medical University Affiliated Kezhou People's Hospital, Kizilsu Kyrgyz Autonomous Prefecture, Xinjiang Uygur Autonomous Region Nanjing Jiangsu China

**Keywords:** emphysematous prostatic abscess, prostatic abscess, transperineal prostate aspiration, transurethral incision of the prostate, transurethral resection of the prostate

## Abstract

Emphysematous prostatic abscess (EPA) is a very rare but fatal urinary tract infection. We retrospectively analyzed the clinical data of a patient with EPA admitted to the First Affiliated Hospital of Nanjing Medical University in January 2022. He experienced EPA progression and deteriorated rapidly after admission, while he recovered immediately after surgical intervention through transurethral internal resection of the prostatic abscess. This case report underscores the importance of early diagnosis and effective treatment for EPA, which can reduce the mortality rate, thereby improving prognosis.

**Summary**

- Emphysematous prostatic abscess (EPA) is a very rare but fatal urinary tract infection.
- CT imaging findings included enlarged prostate volume, low‐density prostatic abscesses, and intraprostatic gas.
- Early diagnosis and effective treatment for EPA can reduce the mortality rate, thereby improving prognosis.

## 1 Introduction

Emphysematous prostatic abscess (EPA) is a very rare but fatal urinary tract infection characterized by a gaseous and purulent exudate in the prostate gland. Susceptible patients often present lower urinary tract obstruction symptoms along with underlying systemic diseases such as diabetes, cirrhosis, or other immune dysfunction, usually occurring between 50 and 60 years old. Common pathogens include *Escherichia coli* , *Klebsiella pneumoniae* , *Proteus mirabilis* , Citrobacter, and yeast [1]. Clinical symptoms are similar to acute prostatitis, such as fever, perineal pain, and dysuria. Digital rectal examination reveals prostate enlargement, fluctuating sensation, and local tenderness in most patients. Since the clinical symptoms and digital rectal examination results are not specific, EPA is further confirmed by imaging examination. CT imaging findings included enlarged prostate volume, low‐density prostatic abscesses, and intraprostatic gas. The disease progresses rapidly and can spread to the urethra, bladder, or rectum after abscess formation, with a high fatality rate if not treated effectively. We retrospectively analyzed the clinical data of a patient with EPA admitted to the First Affiliated Hospital of Nanjing Medical University in January 2022 and searched for relevant literature for summary. Patient data in this study was obtained with the approval of the Medical Ethics Committee of the First Affiliated Hospital of Nanjing Medical University (2024‐QT‐12).

## 2 Patient Data

**FIGURE 1:**
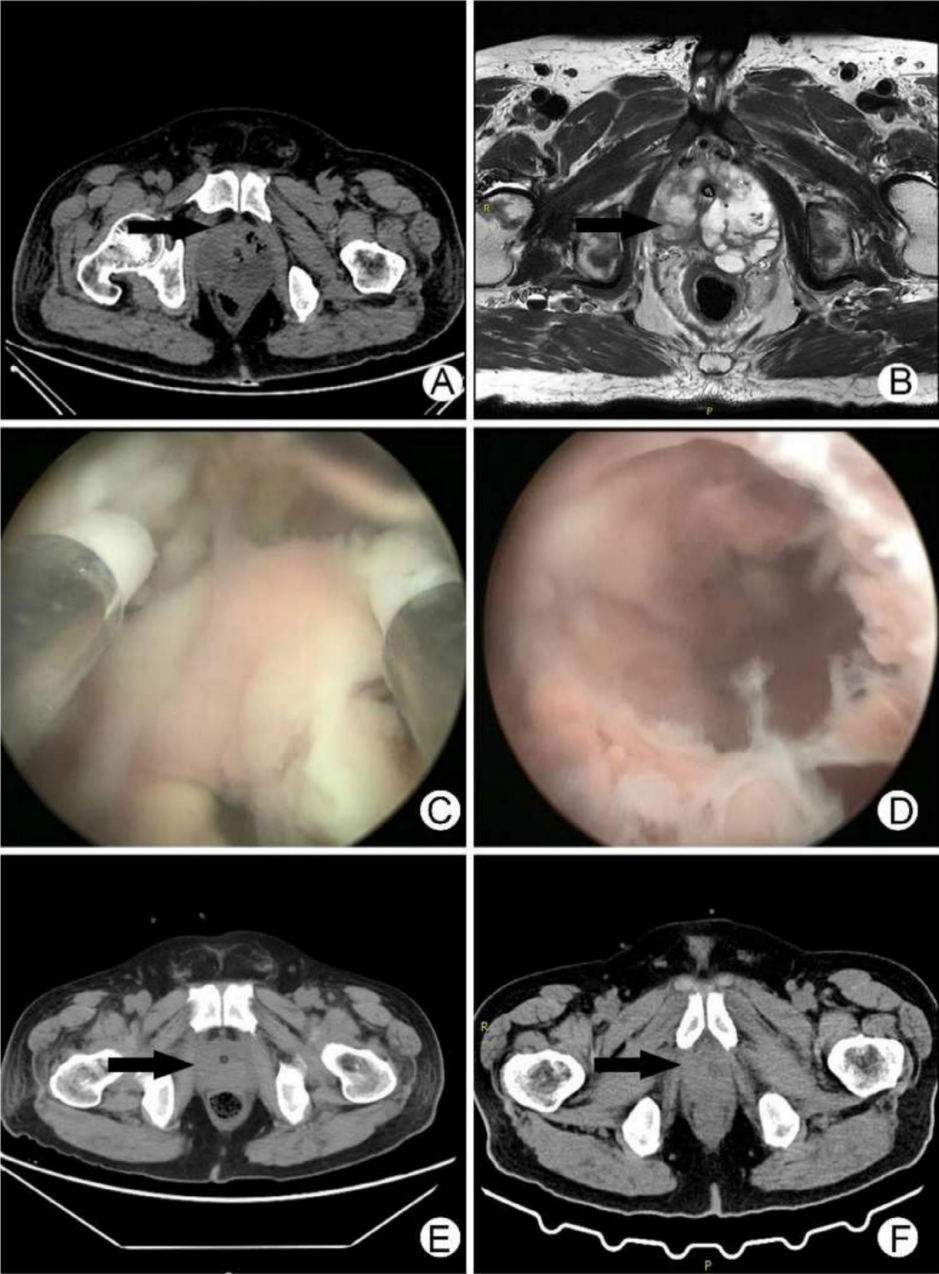
(A) Preoperative CT of the patient showed low‐density prostatic abscesses and intraprostatic gas (arrow position); (B) Preoperative MR of the patient showed prostatic abscess (arrow position); (C) Pink pus was found in the transurethral internal prostatic abscess incision; (D) Large pus of the prostate was found during transurethral internal resection of prostatic abscess; (E) Follow‐up CT performed 2 days postoperatively showed that the prostatic abscess subsided and no intraparenchymal gas (arrow position); (F) Follow‐up CT performed after catheter removal 2 weeks postoperatively showed that the prostatic abscess subsided and no intraparenchymal gas (arrow position).

A 57‐year‐old male patient was admitted to the Department of Urology, the First Affiliated Hospital of Nanjing Medical University on January 22, 2022, due to dysuria, frequent urination, and urgency for 10 years, with symptoms worsening for 1 month. The patient had a history of hypertension and diabetes for 3 years, but he did not pay attention to medication, and he did not monitor blood pressure and blood sugar. 10 days before admission, a B‐ultrasound examination of the urinary system was performed in the emergency department due to dysuria. Results were unclear prostate display, excessive bladder filling, poor bladder lining display, poor internal sound transmission, and infection. The residual urine volume was 973 mL. Thereafter, the patient was treated with indwelling catheterization in the emergency department. A B‐ultrasound was re‐examined 2 days before admission and showed prostatic hyperplasia (53*47*37 mm). The patient had a temperature of 37.5°C and no obvious symptoms of discomfort. After admission, the blood pressure was normal. 2 h after admission, the patient spiked a fever (39.4°C), became exhausted, and had difficulty breathing(30 breaths/min) with no obvious causes, and developed sinus tachycardia(heart rate of 111 beats/min). An immediate peripheral blood glucose test exceeded the upper limit of detection. He received continuous intravenous insulin to control blood sugar and intravenous antibiotic treatment of imipenem 1 g every 8 h along with dexamethasone for fever control. However, his situation deteriorated on the night of admission with changes in consciousness, confusion, and communication difficulties. A complete blood count revealed white blood cells 28.14*10^9^/L, neutrophils 93.9%, red blood cells 3.26*10^12^/L, hemoglobin 100 g/L, hematocrit 27.3%, and platelets 103*10^9^/L. C‐reactive protein was 82.8 mg/L. Procalcitonin was > 100 ng/mL. Urinalysis revealed 3–5 white blood cells/HP, 3–5 red blood cells/HP, 2+ leucocyte esterase, 1+ ketone body, and 4+ urine glucose. Myocardial markers showed high‐sensitivity troponin T 67.46 ng/L and B‐type natriuretic peptide precursor 3651 pg/mL. The patient was placed on oxygen therapy and ECG monitoring, and the condition improved slightly after these supportive treatments. The biochemical results showed urea 32.87 mmol/L, creatinine 496.6 μmol/L, blood sugar 11.76 mmol/L, and albumin 18.9 g/L. Prostate‐specific antigen (PSA) was 2.386 ng/mL, free prostate‐specific antigen (PSA) was 0.145 ng/mL, and urine volume was normal. An abdominal CT scan showed poor bladder filling, exudation around the bladder, prostate volume increased, density decreased, and the left side of the prostate with low‐density abscesses and gas. There were also little perirenal exudation, bilateral prerenal fascia thickened, multiple exudations with fluid accumulation in the adipose space of the lower abdomen and pelvic cavity, and bilateral paracolic sulci fluid accumulation (Figure 1A). According to the CT scan, he was diagnosed with emphysematous prostatic abscess immediately. Yet, he continued to experience persistent high‐grade fever. On the 3rd day of admission, ultrasound‐guided perineal prostatic puncture was performed, and 5 mL of pink pus was extracted and sent for bacterial culture. Prostate MR3T plain scan showed that the prostate volume was approximately 65 mm*51 mm*70 mm; multiple long T1 and T2 signal shadows were seen in the peripheral bands on both sides, with slightly higher DWI and slightly lower ADC values. Multiple long T1 and T2 signal shadows were seen in the left gland, with local protrusion to the rear. Prostatic capsule continuity was satisfactory; bilateral seminal vesicle glands were symmetrical with no abnormal signals (Figure 1B). On the 4th day of admission, considering the patient's severe infection and poor renal function, he underwent suprapubic cystostomy for urine diversion and the urinary tube was removed. However, the patient still presented with repeated high fever. Urine culture result was negative, blood and pus culture results showed *Klebsiella pneumoniae* with ampicillin resistance, imipenem, levofloxacin and other antibiotics sensitive. On the 5th day of admission, the patient's condition deteriorated further, with confusion, passive position, maximum body temperature of 39.4°C, pulse rate 78, blood pressure of 124/72 mmHg, and respiration rate 22. He was scheduled for emergent transurethral internal prostatic abscess incision.

## 3 Surgical Procedure

On the 5th day of admission, the patient underwent transurethral internal resection of prostatic abscess under general anesthesia. After tracheal intubation under general anesthesia, a plasma electroscope was inserted through the urethra to remove both lobes and the middle lobe of the prostate. During the resection, pink pus oozing from the prostatic abscess cavity could be seen, and the resection was continued until the abscess cavity was fully exposed. Large abscess cavities could be seen on both lobes and the middle lobe of the prostate (Figure 1C,D). The pus cavity was fully opened to remove necrotic tissues. The operation time was 40 min, and the intraoperative blood loss was about 50 mL. Postoperatively, a three‐cavity catheter was indwelled with continuous bladder irrigation, and he was transferred to the intensive care unit.

## 4 Results

The patient's situation improved rapidly at POD1. His body temperature dropped to 37.3°C with antibiotic therapy using levofloxacin 0.5 g once a day. On the second day after surgery, he was transferred to the general ward. CT examination reassessment showed that the prostatic abscess subsided significantly, and no gas shadow was observed (Figure 1E). He recovered significantly at POD3. Lab tests revealed 11.85*10^9^/L white blood cells, 85.90% neutrophils, 3.00*10^12^/L red blood cells, 91 g/L hemoglobin, 28.3% hematopoietic volume, and 119*10^9^/L platelets. Other tests revealed procalcitonin 1.33 ng/mL, renal urea 11.61 mmol/L, creatinine 126.9 μmol/L. He was discharged from the hospital at POD3 with continuous oral antibiotics (Figure S1). The urinary tube was removed 2 weeks postoperatively. CT reexamination showed that the prostate area recovered well and no gas shadow was seen (Figure 1F). The vesicostomy tube was removed 4 weeks after operation. He voided well with no frequency, no urgency, no pain, no gross hematuria, and no urinary incontinence. After 2 years of follow‐up, the patient had satisfactory urine control without obvious discomfort.

## 5 Discussion

Various inflammatory and infectious diseases can occur in the genitourinary system, but only about 0.5% of patients with urinary tract infection have prostate abscess [2]. The mortality rate of prostate abscess after effective antibiotic treatment has been greatly reduced. According to the mode of onset, prostate abscess can be divided into primary abscess and metastatic abscess [3]. The primary prostatic abscess is mostly in middle‐aged and elderly people with urogenital tract diseases combined with gram‐negative bacteria infection. Metastatic prostatic abscess is found in severe infection lesions in other parts of the body that transfer to the prostate, and the main pathogen is gram‐positive *Staphylococcus aureus* . Anaerobic bacteria are also pathogenic bacteria of prostate abscess. Prostatic abscesses can spontaneously rupture and spread the infection to the urethra, perineum, bladder, or rectum [4]. Chronic prostatitis and male sterility will appear when the course of the disease is prolonged, and fatal sepsis may occur in some patients with prostatic abscess due to misdiagnosis or inadequate treatment. EPA is a rare but deadly type of prostate abscess, which was first reported by A J Mariani in 1983 [5]. The EPA patient complicated with diabetes was admitted to hospital due to frequent urination, dysuria, and hyperthermia for 3 weeks. The pathogen in urine culture was *Pseudomonas aeruginosa* , and the blood culture was Bacteroides fragilis. 3 days after admission, the disease still progressed after anti‐infection treatment, and the patient was transferred to ICU for treatment. 6 days after admission, venous pyelography showed a gas cloud in the prostate area below the bladder, and the symptoms of urine diversion did not improve after vesicostomy. After incision and drainage in the urethral prostate abscess in the emergency, his condition became stable, but the urethra continued to discharge pus. 20 days after admission, the patient recovered well after transurethral resection of the prostate. The clinical feature of EPA is the formation of abscess in the prostate gland with gas accumulation, and the mortality rate is as high as 25% if the treatment is not timely, even higher than emphysematous cystitis and emphysematous pyelonephritis. Clinical manifestations and signs of EPA vary, including painful urination, fever, frequent urination, urgency, urinary retention, and perineal pain. Since the symptoms are not specific, early diagnosis is difficult for EPA. Imaging techniques such as pelvic CT and transrectal ultrasonography (TRUS) are the most valuable diagnostic tools for early diagnosis of EPA. Pneumogenic pathogens in urogenital tract include *Escherichia coli* , Klebsiella, *Proteus mirabilis* , Citrobacter, and yeast. Reported EPA pathogens include *Klebsiella pneumoniae* , *Pseudomonas* *aeruginosa* , Bacteroides fragilis, Candida albicans, and Bacillus tuberculosis [6, 7].

Abdoulhafid et al. reported a case of EPAs in a patient caused by *Klebsiella pneumoniae* . A 49‐year‐old man with a history of diabetes was admitted to hospital with urinary retention, abdominal pain, and fever. Digital rectal examination revealed tenderness in the prostate area, laboratory examination indicated high inflammatory markers, abdominal CT confirmed an abscess in the right lobe of the liver and multiple lobe abscesses in the prostate area, and the prostate volume was significantly enlarged, about 87 mL. The patient underwent broad‐spectrum antibiotic treatment, strict control of blood glucose, suprapubic cystotomy, and transurethral incision of the prostate abscess, and was discharged from hospital after 3 weeks [8]. EPA induced by *Klebsiella pneumoniae* can also spread and affect the formation of new lesions in other organs, such as endophthalmitis, brain abscess, liver abscess, and psoas major abscess [9]. *Enterobacter cloacae* infection after transurethral resection of the prostate can also cause refractory EPA. The patient in this case was 76 years old and had a history of diabetes with general blood glucose control. Transurethral resection of the prostate was performed due to repeated urinary retention and prostatic hyperplasia. However, the patient developed a high fever and fatigue on the 17th day after surgery. Pelvic CT examination revealed massive gas accumulation in the prostate. Both results of blood and urine culture indicated multidrug‐resistant Enterobacter cloacae infection. Through suprapubic cystotomy, stable blood sugar control, and antibiotic treatment with sensitive drugs, the patient gradually recovered and was discharged from hospital [10].

There may be different clinical manifestations of EPA patients, such as dysuria, frequency, urgency, fever, acute urinary retention, perineal pain, and discomfort in the early stage, and it is difficult to confirm the diagnosis by routine urinalysis and transabdominal prostate ultrasonography, while the formation of a prostatic abscess with gas accumulation can be found by CT or MR examination. Risk factors for EPA include diabetes, bladder outlet obstruction, long‐term indwelling catheterization, history of neurogenic bladder and urethral surgery, liver cirrhosis, and immunosuppression after transplantation. Diabetes combined with urinary tract infection and obstruction may also be the predisposing factor for gas gangrene infection of the urinary system [11]. Undiagnosed diabetes combined with poor glycemic control is an important risk factor for EPA. When symptoms of urinary tract irritation occur in these susceptible people, treatment with conventional anti‐infective drugs is ineffective, and the disease progresses rapidly; the possibility of the disease should be vigilant. Timely pelvic CT scan will help to confirm the diagnosis. It should be noted that a small number of patients with *Klebsiella pneumoniae* liver abscess may present with invasive infection, and when the infection spreads out, pneumonia, pulmonary abscess, pulmonary embolism, pleurisy, peritonitis, subcutaneous abscess, mediastinal infection, spleen abscess, suppurative meningitis, epidural abscess, kidney abscess, prostate abscess, and suppurative arthritis may occur. In clinical diagnosis and treatment, attention should be paid to both the primary focus and metastatic focus of infection [12]. Kundasamy et al. reported a 30‐year‐old male patient with EPA caused by urogenital tuberculosis infection, who was admitted to the hospital due to abdominal pain and dysuria. The left scrotum was significantly enlarged, and local ulcers with skin sinuses appeared. Tuberculosis infection was confirmed by PCR detection of tuberculosis bacilli, and further CT examination indicated EPA. He gradually improved after anti‐tuberculosis treatment [13].

Treatment of EPA is based on adequate abscess drainage and early antibiotic therapy along with strict blood glucose control. Once diagnosed, timely surgical intervention and empirical anti‐infection treatment with broad‐spectrum antibiotics should be added, and sensitive narrow‐spectrum antibiotics should be selected after the results of pathogen culture and drug sensitivity test are reported. Common surgical intervention methods include ultrasound‐guided prostate aspiration, prostate drainage, and transurethral incision of the prostate, which can be selected according to the patient's specific conditions. Gi‐Bum Bae et al. reported a long‐course treatment of an EPA patient infected with *Klebsiella pneumoniae* . The patient was hospitalized due to frequent urination, dysuria, and fever. He had a history of diabetes for 15 years and poor blood glucose control. Emphysematous cystitis was suspected by abdominal plain film and abdominal ultrasound examination in other hospitals. There was no improvement in indwelling catheterization and anti‐infection treatment. The pathogen of urine culture was *Klebsiella pneumoniae* . Further abdominal CT examination and transrectal ultrasound examination confirmed the diagnosis of prostate gas abscess, and the treatment was changed to ceftriaxone + metronidazole + amtronam. On the 23rd day of admission, the urinary tube was removed due to urethral discomfort after suprapubic cystotomy, and another catheter drainage through perineal prostate puncture was performed. His condition improved, and the drainage tube was removed on the 65th day of admission, 4 days after discharge. The cystotomy tube was removed 3 months after the operation [14]. Huai‐Ching Tai et al. reported a 60‐year‐old patient with EPA who was admitted to the hospital due to dysuria, lower abdominal, and perineal pain with fever; the patient had a history of diabetes for 5 years and alcoholic cirrhosis for 6 years. After diagnosis by abdominal CT and rectal ultrasound, the patient underwent transurethral internal resection for prostatic abscess. His symptoms were immediately relieved after surgery without fever, and the catheter was removed after 2 weeks of indwelling, and he recovered well after surgery with smooth urination and no complications [15].

Due to the multilocular abscess of EPA, which contains a lot of gas, thick pus, and necrotic tissue in the pus cavity, it is difficult to fully drain and completely remove the lesion in a single prostate aspiration, and there is a high risk of prolonged healing time and recurrence. Ultrasound‐guided prostate drainage has the advantage of being operable under local anesthesia, which is suitable for elderly people who are generally in poor condition and cannot tolerate anesthesia. However, it has the disadvantages of slow recovery. EPA patients may require multiple drainage tubes, affecting their quality of life. For patients with rapid disease deterioration and progression and stable hemodynamics who can tolerate general anesthesia, transurethral incision of prostate abscess should be considered. Based on the preoperative imaging results displaying the distribution of the pus cavity, all prostate pus cavities should be fully incised to remove the necrotic tissue. Transurethral incision of prostate abscess could provide complete drainage, while having the risk of severe infection. Since the fluid perfusion pressure during urethral incision may result in the expansion of pathogens to the circulatory system, causing severe infection or septic shock. If the lavage pressure is too high, a concurrent vesicostomy could be considered to reduce the risk of postoperative bacteremia and septic shock. It is safer to perform surgery when the patient is hemodynamically stable and suitable for anesthesia. The EPA patient reported here still had rapid disease progression after drug treatment. He finally underwent transurethral incision of prostate abscess, and then his condition rapidly improved, confirming the surgery as an effective treatment. However, one case of successful EPA treatment is unnecessary to formulate a consensus for all EPA patients. Individualized therapy and multidisciplinary care are still warranted for EPA diagnosis and treatment in clinical practice.

## 6 Conclusion

EPA is a rare and relatively dangerous genitourinary infectious disease. Patients with urinary tract infection and suspected prostate abscess combined with diabetes and other potential risk factors should pay attention to EPA's possibility, especially when the patient does not respond to conventional anti‐infection treatment. Early diagnosis, timely, and effective treatment of EPA can reduce mortality with good prognosis.

## Author Contributions

**Xiaomin Yi:** conceptualization, data curation, formal analysis, funding acquisition, investigation, methodology, project administration. **Yunqiang Shao:** data curation. **Jiexiu Zhang:** methodology. **Ruoyun Tan:** funding acquisition.

## Consent

Written informed consent was obtained from the patient to publish this report in accordance with the journal's patient consent policy.

## Conflicts of Interest

The authors declare no conflicts of interest.

## Supporting information


Figure S1.

## Data Availability

The data are available from the corresponding author on reasonable request.
