# Supplementary figures and images for: Emphysematous Prostatic Abscess: A Case Report and Literature Review
Source: Clin Case Rep. 2025 Jun 4;13(6):e70544. doi: 10.1002/ccr3.70544 (PMC12137182; doi:10.1002/ccr3.70544)

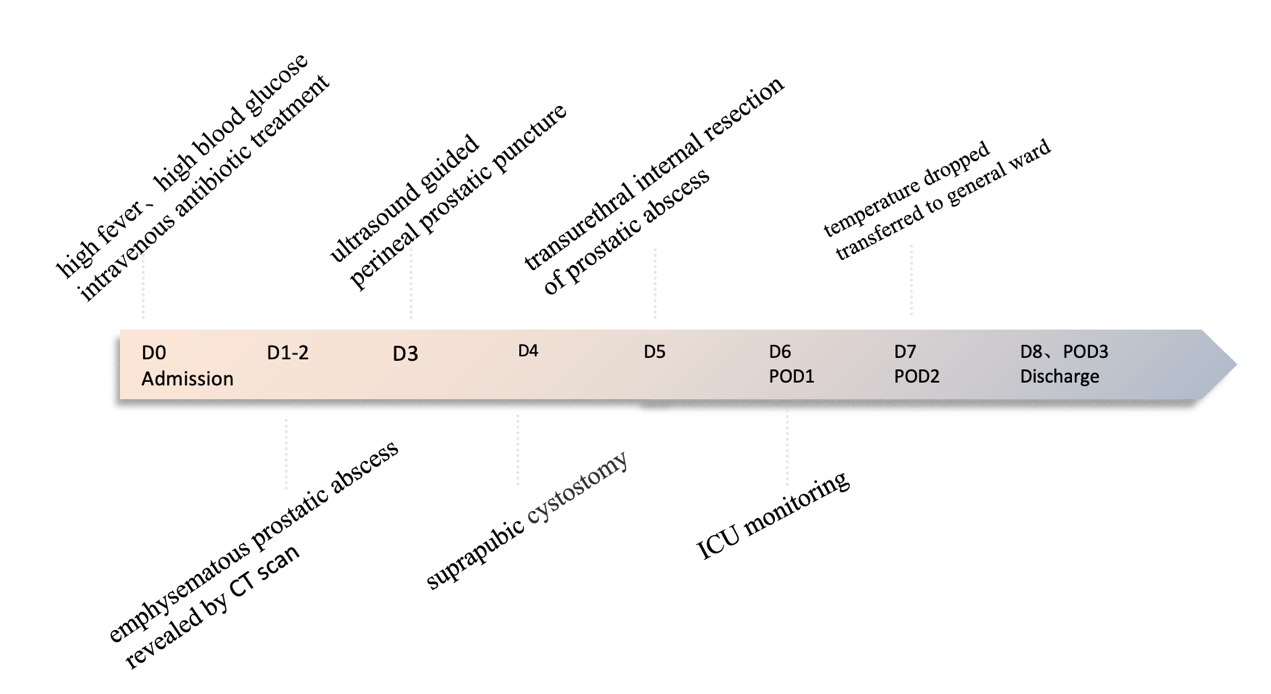


Supplementary figure: Treatment timeline of the EPA patient.

Supplement: Supplementary file 1 — Figure S1. [file CCR3-13-e70544-s001.docx]
